# O06 Inflammatory arthritis triggered by COVID-19

**DOI:** 10.1093/rap/rkaa053.005

**Published:** 2020-11-03

**Authors:** Ghazal Ansari, Frances A Borg, Gouri Koduri

**Affiliations:** Southend University Hospital NHS Foundation Trust, Southend, United Kingdom

## Abstract

**Case report - Introduction:**

COVID-19 is an infectious disease caused by a newly discovered β-coronavirus, named Severe Acute Respiratory Syndrome-Coronavirus-2 (SARS-CoV-2), resulted in a recent pandemic of COVID-19.

As a novel pathogen, the nature and degree of risk of COVID-19 to individuals with rheumatic diseases were unknown, as was its ability to induce musculoskeletal and autoimmune disease. Concerns were related to the chronic autoimmune or inflammatory disease and immune suppressive medications to treat it. The consequences of this infection are currently not fully understood, including the autoimmune sequelae.

Here we present two cases of inflammatory arthritis with a temporal link to COVID-19.

**Case report - Case description: Case 1:**

A 37-year-old Caucasian male was referred to Rheumatology with severe joint pains. He developed flu-like symptoms in early April 2020, with myalgia, fever, sore throat, anosmia, and fatigue. SARS-CoV-2 PCR swab was positive. He recovered from these initial symptoms, however 4 weeks later, he developed pain and swelling in his hands, feet, ankles, and knee joints with early morning stiffness.

On examination, there was marked synovitis of hands, wrists, knees, and ankle joints. Systemic examination was otherwise normal.

**Case 2:**

A 70-year-old lady developed sore throat and cough started in late March 2020. 3 weeks later, she became generally unwell with lethargy and fatigue. Her cough gradually improved, but she continued to experience breathlessness on minimal exertion. In early May 2020, she developed excruciating pain in her hands, wrists, and right knee joints with morning stiffness.

On examination she had synovitis in the wrists, small joints of the hands and right knee. Systemic examination otherwise was unremarkable.

Given the severe inflammatory arthritis, both patients were commenced on oral prednisolone with remarkable improvement 4 weeks later.

**Case report - Discussion:**

We present 2 cases of acute inflammatory arthritis, which were suspected to have been triggered by COVID-19 viral infection without any musculoskeletal complications with good prognosis. COVID-19 is a new disease and our understanding of it is continuing to grow.

The initial concern was that COVID-19 -19 infection may lead to severe illness in immunocompromised patients, including those and with rheumatic conditions. However, this was not seen in large numbers. To our knowledge, COVID-19-related inflammatory arthritis has not previously been reported in the literature.

Our current understanding of the COVID-19 pathogenic mechanisms is limited. However, it is likely that the disease may evolve in overlapping phases.

**Case report - Key learning points:**

In both cases, it was suggested that COVID-19 19 may be a triggering factor for inflammatory arthritis with good prognosis and settled with steroid therapy.

It was suggested that arthritis may occur in patients with COVID-19, in previously fit and well patients without any underlying co-morbidities and autoimmune rheumatic disease and warrants urgent Rheumatology review. However, all COVID-19 suspected cases should be investigated on an individual basis to exclude other diagnosis to avoid missing other common reversible illnesses.

**O06 Table 1:** Investigations at Baseline and 4 weeks

Case 1			Case 2	
	Baseline	4 weeks	Baseline	4 weeks
CRP (<5) mg/L	18	2	276	94
ESR (2-28mm/hour)	3		90	
Hb (130-180 g/L)	143	152	93	114
Wbc (4.0-11.0)109/L	8.0	5.3	11.8	12.1
Neutrophil (1.7-7.5)109/L	5.79	3.28	9.29	10.20
Lymphocyte (1.0-4.5) 109/L	1.39	1.38	1.24	1.14
CK (<200) U/L	90		22	
ANA	Negative		Negative	
ENA	0.2		0.3	
ANCA	Negative		ND	
RF (0-14) U/mL	<10		428	
CCP (0.4-6.9) U/mL	0.8		51	
Immunoglobulins	Normal		IgG,17.9	
Complements	ND		Normal	
CXR	Bil Hilar enlargement		Diffuse widespread air space opacities	
CT Chest	Significant mediastinal Lymphadenopathy with no specific features		Multifocal GGO, patchy consolidation, likely recovery stage of COVID-19

